# Plasma Hypoxanthine-Guanine Phosphoribosyl Transferase Activity in Bottlenose Dolphins Contributes to Avoiding Accumulation of Non-recyclable Purines

**DOI:** 10.3389/fphys.2016.00213

**Published:** 2016-06-08

**Authors:** Roberto I. López-Cruz, Daniel E. Crocker, Ramón Gaxiola-Robles, Jaime A. Bernal, Roberto A. Real-Valle, Orlando Lugo-Lugo, Tania Zenteno-Savín

**Affiliations:** ^1^Programa de Planeación Ambiental y Conservación, Laboratorio de Estrés Oxidativo, Centro de Investigaciones Biológicas del Noroeste, S.C.La Paz, México; ^2^Department of Biology, Sonoma State UniversityRohnert Park, CA, USA; ^3^Instituto Mexicano del Seguro Social, Hospital General de Zona No. 1La Paz, México; ^4^CaboDolphinsCabo San Lucas, Mexico

**Keywords:** bottlenose dolphin, diving, hypoxia, ischemia, purine metabolism, purine salvage

## Abstract

Marine mammals are exposed to ischemia/reperfusion and hypoxia/reoxygenation during diving. During oxygen deprivation, adenosine triphosphate (ATP) breakdown implies purine metabolite accumulation, which in humans is associated with pathological conditions. Purine recycling in seals increases in response to prolonged fasting and ischemia. Concentrations of metabolites and activities of key enzymes in purine metabolism were examined in plasma and red blood cells from bottlenose dolphins (*Tursiops truncatus*) and humans. Hypoxanthine and inosine monophosphate concentrations were higher in plasma from dolphins than humans. Plasma hypoxanthine-guanine phosphoribosyl transferase (HGPRT) activity in dolphins suggests an elevated purine recycling rate, and a mechanism for avoiding accumulation of non-recyclable purines (xanthine and uric acid). Red blood cell concentrations of hypoxanthine, adenosine diphosphate, ATP and guanosine triphosphate were lower in dolphins than in humans; adenosine monophosphate and nicotinamide adenine dinucleotide concentrations were higher in dolphins. HGPRT activity in red blood cells was higher in humans than in dolphins. The lower concentrations of purine catabolism and recycling by-products in plasma from dolphins could be beneficial in providing substrates for recovery of ATP depleted during diving or vigorous swimming. These results suggest that purine salvage in dolphins could be a mechanism for delivering nucleotide precursors to tissues with high ATP and guanosine triphosphate requirements.

## Introduction

**Figure 1:**
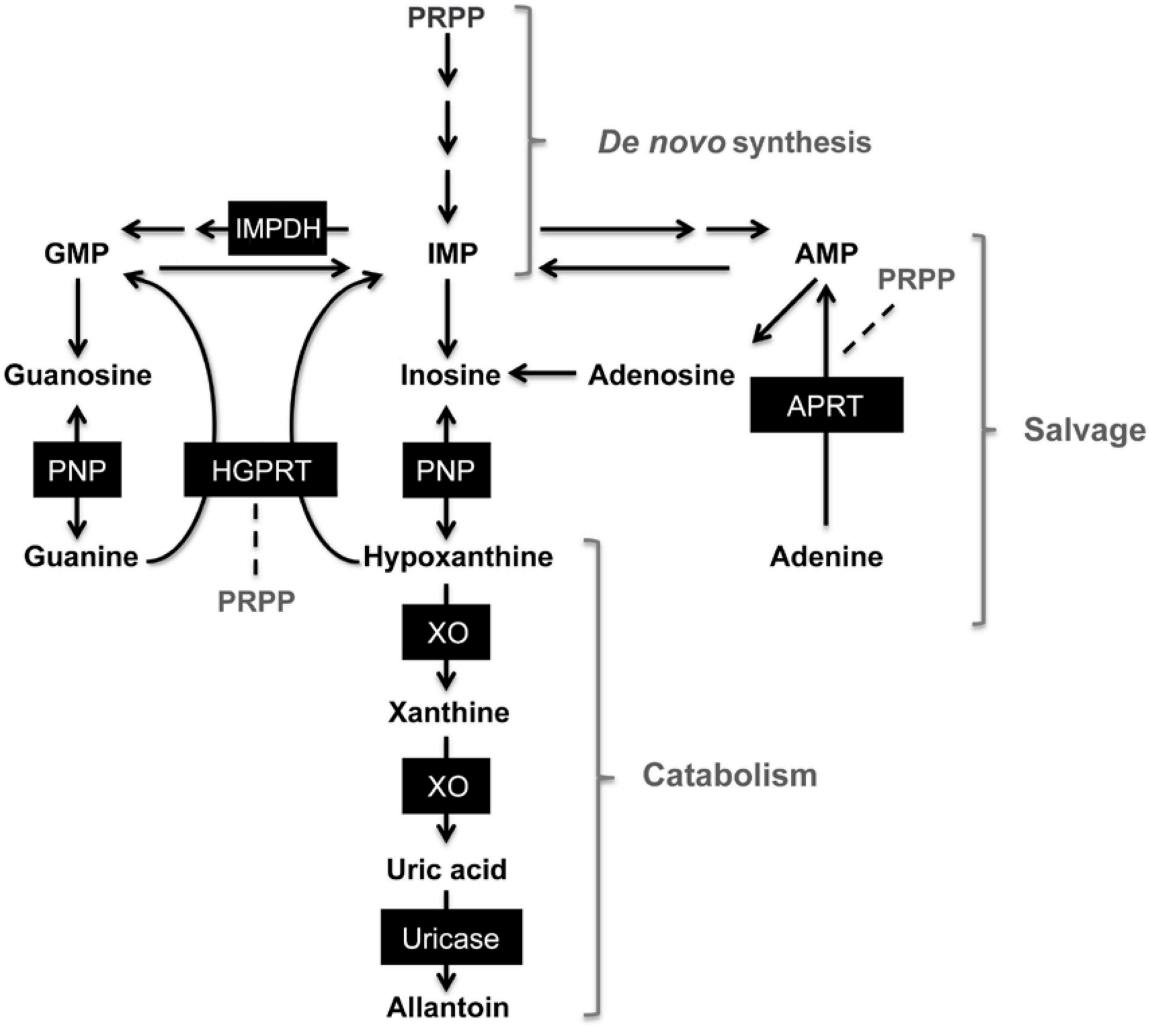
**Simplified scheme of purine metabolism pathways**. 5-phosphoribosyl 1-pyrophosphate (PRPP), inosine monophosphate (IMP), guanosine monophosphate (GMP), adenosine monophosphate (AMP). Dark boxes enclose enzymes involved in *de novo* synthesis, purine salvage and catabolism pathways. Purine nucleoside phosphorylase (PNP), hypoxanthine-guanine phosphorybosil transferase (HGPRT), adenine phosphorybosiltransferase (APRT), xanthine oxidase (XO) inosine monophosphate dehydrogenase (IMPDH). Modified from Torres and Puig (2007).

Purine bases and nucleotides are essential for the appropriate performance of metabolic functions, such as cellular signaling and energy transfer, and as constituents of nucleic acids in all living organisms (Traut, 1994; Baranowska-Bosiacka et al., 2004). Purine metabolism includes closely interrelated synthesis (*de novo*), salvage and catabolism pathways (van den Berghe et al., 2012) (Figure 1). During episodes of ischemia or hypoxia, adenosine triphosphate (ATP) reserves are depleted and hydrolyzed to hypoxanthine (HX); accumulation of HX is associated with reperfusion injury mediated by reactive oxygen species (Rao et al., 1990; Marro et al., 1997). Xanthine oxidase (XO) produces xanthine and uric acid from HX (Nelson and Cox, 2008). Inborn errors of purine metabolism are characterized by abnormal levels of purine metabolites in cells or body fluids, increases or decreases in purine metabolites are related to alterations in activity from enzymes related to purine biosynthesis, purine salvage and purine catabolism (van Gennip, 1999; van den Berghe et al., 2012). Disorders of purine metabolism are related to several clinical signs: arthritis, ataxia, recurrent infections, convulsions, growth retardation, etc. (van den Berghe et al., 2012). Hyperuricemia and gout are among the common pathologies associated to purine disorders, increased synthesis and decreased excretion of uric acid (the final product of purine metabolism in humans) are the main causes of these conditions (Curto et al., 1998). Accumulation of uric acid and decreased activities of enzymes involved in the purine salvage pathway, such as purine nucleoside phosphorylase (PNP) and hypoxanthine-guanine phosphoribosyl transferase (HGPRT), are related with metabolic disorders including hyperuricemia, gout, immunological disorders, neurologic abnormalities and Lesch-Nyhan syndrome, some of which are known to cause early death in humans (Fox, 1981; Curto et al., 1998; Skinner et al., 2006; Torres and Puig, 2007; Jurecka, 2009). In order to avoid uric acid and xanthine overproduction, inhibition of xanthine oxidase activity with allopurinol is recommended (van den Berghe et al., 2012). In non-primate mammals, such as seals and dolphins, the enzyme uricase catalyzes uric acid to allantoin (Skinner et al., 2006).

Marine mammals are exposed to ischemia/reperfusion and hypoxia/reoxygenation cycles during diving as a part of their life history. The physiological adaptations to breath-hold diving in marine mammals have been well described (Kooyman and Ponganis, 1998; Kanatous et al., 2002). In general, tissues from marine mammals have higher capacities to produce superoxide radical as a consequence of ischemia/reperfusion cycles associated to diving (Zenteno-Savín et al., 2002; Vázquez-Medina et al., 2007). However, oxidative damage is avoided, in part due to constitutively higher antioxidant capacities in marine mammal tissues and erythrocytes (red blood cells, RBC) (Wilhelm-Filho et al., 2002; Zenteno-Savín et al., 2012). Purine recycling by inosine monophosphate (IMP)-HGPRT pathway has been suggested in liver and heart from ringed seals after the evidence of HX accumulation caused by ischemia (Elsner et al., 1998). Avoidance of HX accumulation could represent an advantage to reduce reactive oxygen species (ROS) production associated to XO activity. However, knowledge of purine metabolism in these aquatic organisms is still incomplete. Concentrations of HX following experimental ischemia in kidney and heart from ringed seal (*Phoca hispida*) are lower than from domestic pig (*Sus scrofa*) (Elsner et al., 1998). Prolonged fasting increases the expression and activities of HGPRT and XO in northern elephant seals, suggesting an efficient purine recycling and catabolism throughout the fasting period (Soñanez-Organis et al., 2012), which includes frequent occurrence of sleep apnea (Tift et al., 2013). PNP activity in RBC from bottlenose dolphins (*Tursiops truncatus*) is lower than in humans (López-Cruz et al., 2014); therefore, a lower conversion rate from inosine to HX may suggest that bottlenose dolphins avoid reperfusion injury associated to the HX-XO system. Since seals can be considered semiaquatic mammals, we addressed the question of a potential enhancement of purine recycling in a fully aquatic mammal, the bottlenose dolphin, as an adaptation to breath-hold diving. In this study, we compared the principal indicators of purine metabolism pathways between a terrestrial mammal, in which purine disorders are well characterized, and a totally aquatic mammal without previous evidence of purine metabolism enhancements and without prolonged fasting or ischemia conditions.

## Materials and methods

### Chemicals and standards

All the chromatographic standards for purine metabolite determinations were HPLC grade. All solutions were prepared using ultrapure water obtained from a Milli-Q water purification system (Millipore, Bedford, MA, USA). Monobasic potassium phosphate was obtained from Sigma-Aldrich Chemical Co. (St Louis, MO, USA), xanthosine monophosphate (XMP) was purchased from Santa Cruz Biotechnology (Santa Cruz, CA, USA), while acetonitrile (HPLC grade), perchloric acid and potassium hydroxide were obtained from J.T. Baker (Phillisburg, NJ, USA). Potassium carbonate was purchased from Productos Químicos Monterrey (Monterrey, Nuevo Leon, Mexico).

### Instrumentation

Ion-pair high performance liquid chromatography was carried out in a system (Waters 2695, Milford, MA, USA) consisting of an autosampler, quaternary gradient pump solvent management system, on-line degasser and column heater, with a photodiode array detector (PDA, Waters 2996). Chromatography data were acquired and processed using Empower™ software.

### Subjects, sample collection, and handling

Blood samples from 12 human volunteers (8 females, 4 males; 33.1 ± 4.9 years old, 62.6 ± 11.39 kg body mass) were collected. All individuals were informed and oriented regarding clinical procedures, written consent was obtained prior to sample collection. Blood samples were collected from 12 bottlenose dolphins (7 females, 5 males; 9.3 ± 3.9 years old, 190.8 ± 31.5 kg body mass) under human care. Additionally, 8 blood samples from dolphins were collected in order to determine plasma uricase activity. In dolphins, blood sampling procedures require a ventral position and the organisms were at static apnea; sampling procedure lasted between 1 and 5 min. All samples were collected from healthy individuals following an overnight fast. No pregnant or lactating organisms were included in this study. Human samples were obtained at a local hospital (volunteers who attended as altruist donors to the blood bank of the local hospital). Bottlenose dolphin samples were collected at Cabo Dolphins, San José del Cabo and Cabo San Lucas, Baja California Sur, Mexico. Sampling and experimental procedures were reviewed and approved by the local hospital ethics committee and by the Capítulo Baja California Sur de la Academia Nacional Mexicana de Bioética, A.C. Peripheral blood samples were collected as previously reported (López-Cruz et al., 2014) and transported to the laboratory immediately. Plasma was recovered by centrifugation at 1500 × *g* during 5 min at 25°C. RBC were obtained as described by Montero et al. (1995).

### Sample preparation, standards and chromatographic procedures for purine metabolites determination

Purine metabolites were extracted from RBC following the methods described by Giannattasio et al. (2003) with minor modifications. Briefly, RBC were disrupted with cold distilled water (1:6, v/v) and frozen/thawed twice. Intraerythrocytic content was deproteinized with cold perchloric acid (HClO_4_, 0.5 M), shaken vigorously, incubated on an ice bath for 10 min, and centrifuged at 17,900 × *g* for 15 min at 4°C. Potassium hydroxide (KOH, 0.5 M) and potassium phosphate (KH_2_PO_4_, 0.1 M, pH 6.5) were added, and the samples were incubated on an ice bath for 10 min; pH was adjusted to 6–7. Potassium perchlorate was removed by centrifugation at 17,900 × *g* for 15 min at 4°C. Supernatant was filtered (0.22 μM, SLGVR04NL, Millipore) and samples were immediately analyzed. Plasma samples were treated as reported by Stocchi et al. (1987). Briefly, plasma (500 μL) was filtered using a 50 kDa molecular weight filter (Amicon Ultra-4, Millipore) by centrifugation at 2739 × *g* for 15 min at 4°C. The cleared filtered solution was analyzed by HPLC. Standards, solutions and chromatographic procedures were as suggested by Giannattasio et al. (2003) with some modifications. HX, inosine, IMP, NAD^+^, adenosine, adenosine monophosphate (AMP), adenosine diphosphate (ADP), ATP, GDP, guanosine triphosphate (GTP) were dissolved in KH_2_PO_4_ (0.1 M), xanthine and uric acid were prepared in NaOH (40 mM). A mixture including known concentrations of all metabolites was used to prepare a standard curve (1.56–100 μM). A supelcosil LC-18, 150 × 4.6 mm, 3 μm particle size column (Supelco, USA) was used as the stationary phase. Mobile phase consisted of buffer A (100 mM KH_2_PO_4_, 8 mM tetrabutylammonium hydrogen sulfate, pH 6.0) and buffer B (100 mM KH_2_PO_4_, 8 mM tetrabutylammonium hydrogen sulfate with 30% acetonitrile, pH 6.0). Sample analysis was performed using a binary gradient from 100% buffer A to 100% buffer B in a total run time of 25 min at constant flow rate of 1.5 mL min^−1^ at 25°C. A volume of 40 μL was injected of the standard curve and samples. Detection signal was monitored at 254 nm.

### Enzyme activity

The activity of hypoxanthine-guanine phosphoribosyl transferase (EC 2.4.2.8, HGPRT) was measured in plasma and RBC samples by using a PRECICE^®^ HPRT assay kit (NovoCIB, Lyon, France) following manufacturer's instructions. One unit of HGPRT activity is defined as the amount of enzyme that catalyzes the conversion of 1 μM of HX to IMP per minute at pH 8.8 at 25°C. Human recombinant HGPRT was used as a positive control. Results are expressed as nmol h^−1^ mg^−1^ of protein.

The activity of inosine monophosphate dehydrogenase (EC 1.1.1.205, IMPDH) was measured by quantifying the concentration of xanthosine monophosphate (XMP) using HPLC. RBC samples were treated as described by Montero et al. (1995) and assay conditions were as reported by Glander et al. (2001). Briefly, RBC were disrupted with cold distilled water and dithiothreitol (DTT, 1 M), 1:2 v/v, frozen and thawed twice. Assay reaction was started by adding 50 μL of intraerythrocytic content to 120 μL of the reaction mixture (IMP, 1 mM; NAD^+^, 0.5 mM; Na_3_PO_4_, 40 mM; KCl 100 mM; pH 7.4). After 2.5 h of incubation at 37°C reaction was stopped with cold HClO_4_ (0.5 M) followed by vigorously shaking using a vortex. The mixture was centrifuged at 15,800 × *g* during 5 min at 4°C. Supernatant was transferred to a clean tube and neutralized with potassium carbonate (K_2_CO_3_, 0.5 M), frozen at −80°C for 30 min, thawed, centrifuged, and finally filtered through 0.22 μM membrane prior to injection into the HPLC. Results were expressed in μmol h^−1^ mg^−1^ of protein. One unit of IMPDH activity is defined as the amount of enzyme necessary to catalyze the oxidation of 1 μM of IMP to XMP per min at 37°C. Chromatographic procedures for IMPDH activity determination were only minimally modified (acetonitrile was replaced for methanol) from the HPLC conditions described above for purine metabolites quantification. Stationary phase consisted of a Hypersil 125 × 4.6 mm, 3 μm particle size column (Thermo Scientific, USA). Mobile phase consisted of a binary gradient from 100% Buffer A (KH_2_PO_4_, 100 mM with 8 mM tetrabutylammonium hydrogen sulfate, pH 6.0) to 100% Buffer B (KH_2_PO_4_ with methanol 30%), in a total run time of 26 min at constant flow rate of 1.2 mL per min. A standard curve of XMP (0.39–50 μM) was used to calculate IMPDH activity. Detection signal was monitored at 254 nm. Peak identification was confirmed by retention time, absorption spectrum and whether the sample showed overlapping peaks following enrichment with the standard.

Additionally, 8 samples of plasma from dolphins were collected in order to determine uricase activity. Uricase (EC 1.7.3.3) activity was measured by fluorescence in a microplate reader (Synergy 4, BioTek) using the Amplex Red Uric Acid/Uricase Assay kit (Molecular Probes, Eugene, OR) according to the manufacturer's instructions. One unit of uricase is defined as the amount of enzyme necessary to convert 1 μmole of uric acid to allantoin per minute at pH 8.5 and 25°C.

### Total soluble protein content

All data were standardized to mg of protein. Total soluble proteins were quantified as described by Bradford (1976) using a commercial kit adapted for use in a microplate reader (Bio-Rad Laboratories, Hercules, CA, USA). Bovine serum albumin (BSA) was used as standard, and a standard curve (0.005–0.2 mg mL^−1^) was used for calculations. Results were expressed in mg of protein mL^−1^.

### Statistical analyses

Data were grouped by species. Normality and homoscedasticity of the data were tested using Kolmogorov–Smirnov and Levene tests, respectively. Given the non-normal distribution of the data, significant differences were determined with Mann–Whitney *U*-tests (Zar, 2009). All statistical analyses were performed using Statistica^®^ 6.0 and SPSS v22 (IBM, Armonk, NY, USA). Statistical significance was considered when *p* < 0.05. Data are presented as median and 25th–75th percentiles.

## Results

### Concentration of purine metabolites

The concentration of purine metabolites was determined to elucidate purine requirement differences in RBC and plasma from dolphins and humans. These results are summarized for RBC in Table 1 and for plasma in Table 2. In RBC from humans HX, IMP, NAD^+^, AMP, ADP, GTP, ATP were detected; in addition to these, GDP was identified in RBC from dolphins. Determination of HX, xanthine, uric acid, IMP, adenosine, NAD^+^, AMP, ADP, GDP, GTP, and ATP was achieved in plasma of both species. Adenosine levels in RBC of both species were below the detection limit (0.39 μM mL^−1^). In RBC from dolphins the concentrations of NAD^+^ (*U* = 0.001, *p* < 0.01) and AMP (*U* = 12.0, *p* < 0.01) were higher than in humans. HX (*U* = 20.0, *p* < 0.01), ADP (*U* = 27.0, *p* < 0.01), GTP (*U* = 21.0, *p* < 0.01), and ATP (*U* = 7.0, *p* < 0.01) concentrations were higher in RBC from humans than in dolphins. Higher HX (*U* = 32.0, *p* = 0.02) and IMP (*U* = 26.0, *p* = 0.049) concentrations were found in plasma from dolphins than in humans. Plasma ADP (*U* = 5.0, *p* < 0.01), GTP (*U* = 27.0, *p* < 0.01), and ATP (*U* = 19.0, *p* < 0.01) concentrations were lower in dolphins than in humans. Plasma uric acid concentration was >16-fold lower in dolphins than in humans (*U* = 0.001, *p* < 0.01).

**Table 1:** **Purine metabolite concentrations (μmol mg^−1^ of protein) in erythrocytes (RBC) from humans and bottlenose dolphins (*n* = 12 for each species)**.

	**Human (μmol mg^−1^ of protein)**	**Bottlenose dolphin (μmol mg^−1^ of protein)**
HX	0.18 (0.11−0.24)^*^	0.057 (0.051−0.084)
IMP	0.07 (0.05−0.72)	0.18 (0.12−0.27)
NAD^+^	0.004 (0.003−0.006)	0.09 (0.05−0.12)^*^
AMP	0.08 (0.07−0.17)	0.37 (0.23−0.57)^*^
GDP	–	0.06 (0.05−0.07)
ADP	0.31 (0.28−0.37)^*^	0.24 (0.19−0.28)
GTP	0.06 (0.06−0.09)^*^	0.04 (0.03−0.05)
ATP	0.84 (0.45−0.92)^*^	0.09 (0.05−0.15)

Data are presented as median (25th-75th percentiles).

* Significant differences between species, p < 0.05.

HX, hypoxanthine; IMP, inosine monophosphate; NAD^+^, nicotinamide adenine dinucleotide; AMP, adenosine monophosphate; GDP, guanine diphosphate; ADP, adenosine diphosphate; GTP, guanine triphosphate; ATP, adenosine triphosphate.

**Table 2:** **Purine metabolite concentrations (μmol mg^−1^ of protein) in plasma from humans and bottlenose dolphins (*n* = 12 for each species)**.

	**Human (μmol mg^−1^ of protein)**	**Bottlenose dolphin (μmol mg^−1^ of protein)**
HX	0.07 (0.04−0.14)	0.33 (0.22−0.54)^*^
Xanthine	0.28 (0.21−0.48)	0.22 (0.14−0.24)
Uric acid	7.01 (4.71−9.28)^*^	0.44 (0.24−0.76)
IMP	0.12 (0.09−0.18)	0.25 (0.15−0.38)^*^
Inosine	0.15 (0.09−0.26)	0.12 (0.09−0.17)
NAD^+^	0.12 (0.07−0.18)	0.17 (0.10−0.25)
Adenosine	0.04 (0.02−0.11)	0.016 (0.015−0.017)
AMP	0.06 (0.05−0.12)	0.05 (0.04−0.06)
GDP	0.04 (0.04−0.05)	0.04 (0.03−0.05)
ADP	0.07 (0.06−0.10)^*^	0.03 (0.02−0.04)
GTP	0.05 (0.04−0.069)^*^	0.04 (0.030−0.04)
ATP	0.04 (0.03−0.05)^*^	0.02 (0.01−0.03)

Data are presented as median (25th-75th percentiles).

* Significant differences between species, p < 0.05.

HX, hypoxanthine; IMP, inosine monophosphate; NAD^+^, nicotinamide adenine dinucleotide; AMP, adenosine monophosphate; GDP, guanine diphosphate; ADP, adenosine diphosphate; GTP, guanine triphosphate; ATP, adenosine triphosphate.

### Enzyme activities

The data of HGPRT and IMPDH activities in RBC are summarized in Table 3. HGPRT activity was measured by its central role to recycle nucleotides through the salvage pathway. The activity of HGPRT in RBC from dolphins was lower than in humans (*U* = 0.001, *p* < 0.01). In RBC from dolphins, a low HGPRT activity is apparently sufficient to recycle HX to IMP, since IMP concentration was not different from humans. In plasma from dolphins, HGPRT activity was 3.25 (1.23–3.95) nmol h^−1^ mg^−1^ of protein and may indicate a role to balance or deliver purine requirements to tissues/organs committed to hypoxia/ischemia associated to diving. The activity of HGPRT was below the detection limit (6.75 nmol mL^−1^ h^−1^) in 58.33% of human plasma samples [0.33 (0.260–1.899) nmol h^−1^ mg^−1^ of protein], and in three of these samples, was close to the value obtained for blanks. IMPDH represents a rate-limiting enzyme to guanine nucleotide biosynthesis in the *de novo* pathway. There were no statistically significant differences in RBC IMPDH activity between dolphins and humans. This suggests that there is no significantly higher contribution of *de novo* pathway to modulation of purine metabolism in dolphins than in terrestrial mammals. Uricase activity was determined in plasma from dolphins in order to test if this enzyme directly influenced lower levels of xanthine and uric acid. There was no evidence of uricase activity.

**Table 3:** **Enzyme activity of hypoxanthine-guanine phosphorybosil transferase (HGPRT, nmol h^−1^ mg^−1^ of protein) and inosine monophosphate dehydrogenase (IMPDH, μM h^−1^ mg^−1^ of protein) in erythrocytes (RBC) from humans and bottlenose dolphins (*n* = 12 for each species)**.

	**HGPRT activity (nmol h^−1^ mg^−1^ of protein)**	**IMPDH activity (μmol h^−1^ mg^−1^ of protein)**
Human	123.04 (102.17−140.99)^*^	0.032 (0.024−0.041)
Bottlenose dolphin	41.81 (33.73−45.36)	0.021 (0.13−0.036)

Data are presented as median (25th-75th percentiles).

* Significantly different p < 0.05.

## Discussion

Evidence suggests an enhancement to recycle purines under circumstances like ischemia and prolonged fasting in seals. For instance, ringed seals possess the ability to maintain HX levels below terrestrial mammals under ischemic conditions (Elsner et al., 1998) and northern elephant seals increase purine recycling and catabolism during prolonged fasting (Soñanez-Organis et al., 2012). Further, purine metabolism modulation is species-specific, even among diving mammals (López-Cruz et al., 2014). We measured the concentration of purine metabolites relating to several steps of purine pathways, HGPRT as indicator of purine salvage pathway and IMPDH as indicator of *de novo* biosynthesis in dolphin plasma and RBC. In northern elephant seals, prolonged fasting increases plasma HGPRT activity (Soñanez-Organis et al., 2012). Similarly, in this study, plasma HX and IMP concentrations (i.e., HGPRT substrate and product, respectively) were significantly higher in dolphins than humans. This suggests an efficient mechanism to prevent two detrimental processes in dolphins; namely, (1) accumulation of non-recyclable purines (xanthine, uric acid), and (2) overproduction of ROS (mediated by increased XO activity as a consequence of ischemia/reperfusion and hypoxia/reoxygenation cycles associated to diving). HGPRT activity in plasma of dolphins could offer an advantage to salvage and supply nucleotide precursors to cells or tissues that require returning to basal levels following an episode of ischemia and/or hypoxia. For example, it is possible that under ischemic/hypoxic conditions, nucleosides and oxypurines are released to plasma, a fraction of purines are delivered directly to RBC to produce new nucleosides and nucleotides (Dománski et al., 2012). High permeability of RBC from bottlenose dolphins and humans to nucleosides and glucose (Craik et al., 1997) could ensure suitable levels of adenine and guanine nucleotides inside these cells. To our knowledge, plasma HGPRT activity has not been previously reported in humans. A specific search for similar publications in health area databases Pubmed, EBSCO, Springer Link and ScienceDirect was performed on May 20th, 2015 using the following keywords “hypoxanthine-guanine phosphoribosyl transferase,” “activity,” “human plasma.” The displayed results were 657 publications from the year 1980 to 2015; however, none of these publications report HGPRT activity in human plasma samples. At this time, we cannot discard HGPRT activity levels detected in human plasma samples being associated to hemolysis.

Purine metabolism in human RBC is apparently limited to salvage and catabolism pathways since the *de novo* pathway is not active in these cells (Lowy and Dorfman, 1970; Skotnicka et al., 2008; Dudzinska et al., 2010). However, IMPDH activity has been reported in RBC (Montero et al., 1995). Intraerythrocytic activity of PNP, an enzyme related to purine salvage, is lower in marine mammals (dolphins and northern elephant seals) than in terrestrial mammals (Craik et al., 1997; López-Cruz et al., 2014). In this study, intraerythrocytic HGPRT activity was lower in dolphins than in humans. These results suggest that purine recycling in RBC from dolphins is lower than in humans. Lower HX and higher (NS) IMP concentrations in RBC from dolphins suggest an efficient reutilization of purines via the HGPRT-IMP pathway than in humans. Even though ATP and ADP concentrations were lower in RBC from dolphins, AMP concentrations were higher than in humans. It is possible that in dolphins, ATP degradation could be modulated in order to avoid accumulation of non-recyclable purines (i.e. xanthine and uric acid) and to optimize ATP reserves. In accordance with this suggestion, lower HX accumulation has been reported in ringed seal kidney and heart than in the same tissues of domestic pig in response to *in vitro* ischemia (Elsner et al., 1998). In RBC from dolphins in this study, ATP conservation was not evident since ATP concentration was significantly lower than in humans. At this time, we cannot discard low ATP and ADP concentrations in RBC from bottlenose dolphins being a consequence of breath-hold diving, since during blood sampling dolphins were at static apnea. It is possible that such apneic bouts are tantamount to preconditioning and which could lead to reduced content of purine metabolites. Furthermore, in humans, RBC ATP is not recycled from HX since humans lack the enzyme adenylosuccinate synthetase (Simmonds et al., 1989; Schuster and Kenanov, 2005). The activity of IMPDH, a rate-limiting enzyme for guanine nucleotide metabolism (Ignoul and Eggermont, 2005), was not different between humans and dolphins. Thus, recycling by the salvage pathway, taking advantage of preformed purines seems to be sufficient to maintain the pool of guanine nucleotides in both species. GTP levels are considered relatively low in RBC from humans (Simmonds et al., 1989). IMPDH activity in healthy subjects has been reported in the range of 4–183 pmol h^−1^ mg^−1^ of protein (Montero et al., 1995).

Accumulation of non-recyclable purines is of clinical importance in humans. There was no significant difference in plasma xanthine concentration between humans and dolphins; however, uric acid content was >16-fold higher in humans. Considering plasma HGPRT activity, HX and IMP concentrations, we suggest that dolphins recycle purines at a higher rate than humans. However, a special consideration should be made for the catabolism pathway. Evolutionary divergence of nitrogen compound elimination could contribute to uric acid concentration differences since this is the final product of purine catabolism in primate mammals. In marine mammals the final product is allantoin (Skinner et al., 2006). Since no uricase activity was found in plasma from dolphins, purine recycling pathway appears to play an essential role to avoid purine over-accumulation. Further, the antioxidant role of uric acid may convey an advantage to the observed levels of this by-product in human plasma (Ames et al., 1981). Uric acid is responsible of 60–70% of the antioxidant capacity in plasma from humans (Proctor, 2008). Purine metabolism is highly influenced by diet. In dolphins from managed populations, protein- and purine-rich diets seem to predispose ammonium urate nephrolithiasis (Smith et al., 2014). Purine recycling in plasma from wild dolphins should be investigated to compare differences in recycling rates between organisms with diverse diets.

In a previous study, the activity of two of the enzymes related to purine metabolism, PNP and XO in the same dolphin population was analyzed (López-Cruz et al., 2014). No differences between XO activity in plasma from humans and dolphins were found; however, we did find lower PNP activity in RBC from dolphins. Our results for PNP activity are in agreement with those reported in the literature (Sandberg et al., 1955; Craik et al., 1997) and could in part explain the lower HX levels observed. Since a previous report suggested rapid entry of adenosine into dolphin erythrocytes (Craik et al., 1997), and given that further information is not available, at this time we cannot discard the possibility that other purine nucleosides, such as HX, could be similarly transported in RBC to/from tissues.

ATP-binding cassette transporters could contribute to maintain homeostasis in dolphin RBC. Literature on purine transporters in marine mammals is scarce. Craik et al. (1997) examined nucleoside transport in erythrocytes from bottlenose dolphins and reported functional properties similar to those of *es* transporters in human RBC. Even if HX is taken outside the erythrocytes, inosine is not a source of energy in RBC from bottlenose dolphins and PNP is lower in comparison with pigs and humans (Craik et al., 1997); under circumstances of ATP depletion such as diving or exercise HX levels could be delivered to RBC, to take advantage of preformed purines and recover energy levels rapidly; this has been suggested for horses (Jarvis and Harris, 1998).

In conclusion, these results indicate a specific purine metabolite modulation in plasma from dolphins, particularly for avoiding accumulation of non-recyclable purines. Considering RBC are highly permeable but are not highly metabolically active and that purine metabolism has been shown to be tissue-specific (Soñanez-Organis et al., 2012), in dolphins the circulating HGPRT-IMP pathway may contribute to providing purine nucleosides and nucleotides to other cells or tissues, particularly to those with high ATP and GTP requirements. Enhancement of circulating purine salvage pathway could provide a mechanism for rapid replacement of nucleotides to other cells or tissues depleted during diving or swimming in bottlenose dolphin. Plasma HGPRT activity in bottlenose dolphins suggests an efficient mechanism for avoiding accumulation of non-recyclable purines. The results from this study suggest that purine recycling, in addition to contributing to reestablish important nucleobases concentrations, may provide an additional mechanism for marine mammal tissues to prevent ROS overproduction and to avoid oxidative damage after routine diving cycles. Further investigation of purine *de novo* synthesis, salvage and catabolism pathways in other tissues in marine and terrestrial mammals is required.

## Author contributions

TZ group and research coordinator, PI; DC co-PI; RG sample collection, data interpretation; JB and RR dolphin caregivers, sample collection; OL technical support, sample analysis, RL grad student, wrote first draft of manuscript, sample collection and analyses, data analyses and interpretation.

### Conflict of interest statement

The authors declare that the research was conducted in the absence of any commercial or financial relationships that could be construed as a potential conflict of interest.
